# Ferric perchlorate hydrate as a new catalyst for highly efficient esterification of cellulose at room temperature

**DOI:** 10.1038/s41598-022-09669-w

**Published:** 2022-04-04

**Authors:** Safaa Ragab, Ahmed Eleryan, Ahmed El Nemr

**Affiliations:** National Institute of Oceanography and Fisheries, NIOF, Kayet Bey, El-Anfoushy, Alexandria, Egypt

**Keywords:** Chemistry, Materials science

## Abstract

Ferric perchlorate was tested for the first time as a new catalyst to accelerate the esterification of microcrystalline cellulose (MCC) at room temperature in a less amount of acetic anhydride compared to the amount used in the conventional methods. It was possible to manufacture cellulose acetate (CA) with a high yield of up to 94%. The influence of changes in reaction time, catalyst amounts, and acetic anhydride on the characterization of cellulose acetate produced was investigated. The optimum condition for esterification of 2.0 g (12.34 mmol) MCC was found to be: 10 mL (105.98 mmol) AC_2_O, 200 mg (0.564 mmol, anhydrous basis) of Fe(ClO_4_)_3_·xH_2_O and 1 h reaction time at room temperature. The substitution degree of CA was investigated by FTIR and ^1^H-NMR spectroscopy. Thermal stability of CA was studied using TGA, DTA and DSC analyses. The degree of polymerization and the polydispersity index (PDI) were obtained using Gel permeation chromatography (GPC). This study verified the direct and efficient synthesis of di- and tri-cellulose acetate in one–pot reaction using Fe(ClO_4_)_3_·xH_2_O as a catalyst without using solvent.

## Introduction

This work is a part of a research topic that continues to give much attention about the use of catalyst materials for one-pot organic reactions. Ester is widespread in nature and is also widely used in industry and cellulose acetate being the most popular ester products^1,2^*.* The most important cellulose ester is cellulose acetate, which is a nonirritant, biodegradable for many degree of substitution and some cellulose acetates are nontoxic and water insoluble cellulose derivative^3,4^. Cellulose acetate is a partially acetylated cellulose with an acetyl content ranging from 29.0 to 44.8%, which is comparable to mono, di-, and tri-acetate. Plastics, films, photographic, lacquers, fabrics, and dialysis or reverse osmosis membranes are only a few of the industrial uses of cellulose acetate. Furthermore, cellulose acetate is used to coat tablets with semipermeable coatings, especially in osmotic pump-type tablets and microparticles for controlled drug release^5–8^. Cellulose acetate, has been used in electrophoresis as a mean of separating the lipoprotein classes^9^. Cellulose acetate is the most commonly used and tested material for natural gas sweetening^10^.

From an economic and environmental standpoint, conducting one–pot reactions at room temperature under solvent-free conditions and using heterogeneous catalysts have become increasingly important in recent years. In general, the catalyst must possess a number of characteristics, including low cost, ease of handling and transportation, and high effectiveness. Ferric perchlorate is extremely useful in organic chemistry because it is cheap, readily accessible, and effective for a variety of purposes requiring Lewis acid catalysis^11–13^. Ferric perchlorate has been used to form and cleave carbon–oxygen bonds in ethers, esters, alcohols, epoxides, tetrahydropyranyl ethers, and acetals due to its versatility^11,14^.

In one pot, primary and secondary benzylic alcohols with nitriles were converted to various substituted amides using an efficient reagent, ferric perchlorate hydrate supported on silica gel^15^. It's worth noting that Fe(ClO_4_)_3_·xH_2_O does not need a stoichiometric amount of base because, unlike all other metals, it can form the dionato chelate complex without deprotonation and even in Brønstedt acidic media^16,17^. Ferric perchlorate has been used in the creation of carbon–carbon bonds. The ferric perchlorate catalyzed Michael reaction of *β*-esters with methyl vinyl ketone and methyl acrylate as acceptors yielded products in 99% yield after the catalyst was removed by simple filtration^17^. Ferric perchlorate was successfully used to oxidize Hantzsch 1,4-DHPs^11,18^. Oximes were also converted into aryl hydrazones in the presence of Fe(ClO_4_)_3_ in 1,2-dichloroethane^19^. Recently, El Nemr and his coworkers have reported *N*-iodosuccinimide (NIS), I_2_^12^, FeCl_3_^21^, Zncl_2_^22^_,_ MnCl_2_^23^_,_ ZrOCl_4_^24^ and NiCl_2_^25^ as Lewis acid catalysts in esterification of cellulose.

The findings of cellulose acetate synthesis published in the literature showed a long reaction time, the use of an excess of acetic anhydride, and high temperature application, all of which may increase the cost-effectiveness. As a result, we hypothesized that using less acetic anhydride and using room temperature as the reaction temperature for the development of cellulose acetate would be advantageous in terms of reaction simplicity and product properties. As a result, in previous work experiments, we attempted to test various catalysts for cellulose acetate synthesis. Based on these hypothesis, the aim of this work is to demonstrate for the first time the use of Ferric perchlorate hydrate as an efficient and green catalyst for the synthesis of cellulose acetate under solvent-free conditions. In the presence of acetic anhydride at room temperature, ferric perchlorate was successfully used in a catalytic amount to transform microcrystalline cellulose directly to the corresponding acetates with various degrees of substitution in excellent yields in a one-pot reaction. The prepared cellulose acetate was characterized using FTIR, TGA, DTA, DSC, NMR, and GPC.

## Material and methods

### Materials

Microcrystalline cellulose (MCC) was procured from Chemieerzeugnisse und Adsorptions technique AG. Switzerland. Fluka analytical provided acetic anhydride and ethyl alcohol. Ferric perchlorate hydrate (Fe(ClO_4_)_3_·xH_2_O) was purchased from Merck.

### Method

In a round flask (100 mL), 2.0 g (12.34 mmol) of MCC was combined with varying amounts of acetic anhydride [10 (105.98 mmol), 12 (127.18 mmol), and 16 (169.58 mmol) mL], and then the catalyst ferric perchlorate hydrate was applied in four different amounts [50 (0.141 mmol), 100 (0.282 mmol), 200 (0.564 mmol) and 300 (0.847 mmol) mg]; the catalyst should be added to the acetic anhydride before the addition of the MCC to maintain the reaction temperature constant at room temperature). The reaction mixture was then stirred at room temperature for different period of time (0.5, 1, 2, 3, and 4 h). After the reaction time was completed, about 10 mL of ethyl alcohol was added drop by drop, followed by 100 mL of distilled water, and the mixture was allowed to precipitate for 1 h. The white precipitate was filtered out and washed several times with distilled water before being washed with a small amount of 70% ethanol (10 mL). The products were obtained by drying the wet precipitate for 24 h at 50 °C in a drying oven and then weighing it^7,8,12,20–23^.

### Characterization

To analyze the functional groups, all of the products were characterized using a Bruker FTIR Model Vertex 70 spectrometer coupled to an ATR unit in the spectral range of 4000–400 cm^–1^. The Jeol Nuclear Magnetic Resonance Spectrometer 500 MHz was used to obtain ^1^H-NMR in CDCl_3_. Infrared spectrometry was used to assess the DS values of the cellulose acetates, which were then confirmed using ^1^H-NMR spectrometry. The molecular weight, degree of polymerization, and polydispersity of a substance were also calculated using gel permeation chromatography (GPC) (Agilent Technologies-1260 infinity II series) using tetrahydrofuran 1.0 mL/min as mobile phase at room temperature^7^. Thermogravimetric analysis (TGA, DSC, and DTA) was carried out with the SDT650-Simultaneous Thermal Analyzer instrument in the temperature range of room temperature to 900 °C, with a ramping temperature of 5 °C per minute under atmosphere of nitrogen gas (100 mL/min).

### Determination of DS

The degree of substitution (DS) of CA was identified experimentally and confirmed theoretically by integrating the areas of FTIR peaks and verified by integrating the areas of ^1^H-NMR peaks as previously stated^7,8,12,20–23,26–28^.

## Results and discussion

**Scheme 1:**
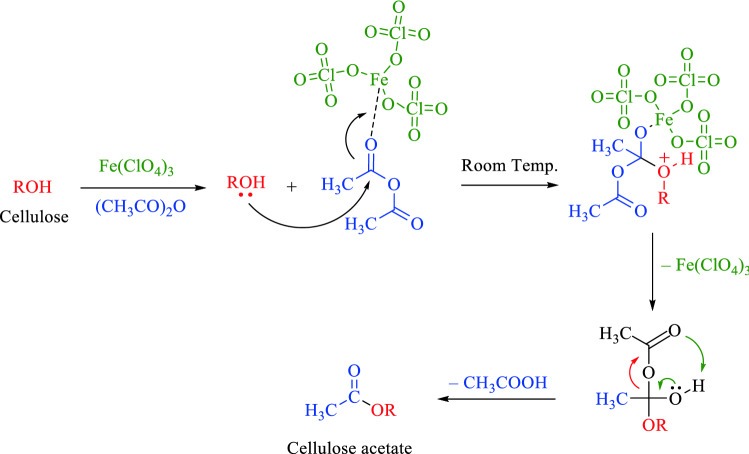
Acetylation mechanism of MCC by using acetic anhydride and ferric perchlorate as catalyst^7,8,12,20–23^.

In this paper, we expedited direct esterification of MCC to their corresponding cellulose acetates using acetic anhydride containing various amounts of inexpensive and available Fe(ClO_4_)_3_·xH_2_O as a new catalyst for cellulose acetylation. From our point of view, this reaction takes place through ferric perchlorate which plays an important role as Lewis acid catalyst where it accelerates the acetylation process by activating the acetyl portion of the acetic anhydride, then facilitates attacking the oxygen atom of the cellulose by the electron pairs on it and then allows the loss of the acetic acid molecule to complete the acetylation process (Scheme 1)^12,14,20,22^.

Initially, a routine study for direct esterification of MCC with catalytic amount of ferric perchlorate hydrate (50 mg) in various amounts of acetic anhydride (10, 12, and 16 mL) and reaction times (0.5–4 h), was carried out at room temperature. However, all samples of micro crystalline cellulose were easily converted to acetylated cellulose (Samples **1**–**15**) by that method (Table 1). Interestingly, in the course of this work, we have noticed that such a reaction proceeds optimally by increasing the time reaction from 0.5 to 4 h not by increasing the amount of AC_2_O from 10 to 16 mL (Table 1). Sample **3** reflects ideal conditions for preparing cellulose di-acetate as a final product where the percentage yield is 87.32% (DS_exp_, 2.62) (cellulose di-acetate typically has a DS range of 2.4 to 2.6, while DS over 2.7 should be referred to as cellulose tri-acetate) (Table 1). The proposed method showed that we are able to control the synthesis of cellulose di-acetate and cellulose tri-acetate by controlling the amount of catalyst, acetic anhydride and reaction time without the use any additional solvent such as ionic liquid solvent (Table 1). Therefore the cellulose di-acetate was directly prepared without the need for back hydrolysis from the cellulose tri-acetate species, which is typically required for industrial applications.

**Table 1:** Acetylation of MCC (2.0 g) using acetic anhydride in presence of Fe(ClO_4_)_3_·xH_2_O (50, 100, 200, and 300 mg) at room temperature and different times (0.5–4 h).

Sample no	Fe(ClO_4_)_3_ (mg)	Ac_2_O (mL)	Contact time (h)	CA yield (g)	Yield % of CA	DS_Exp_	DS_FTIR_	Solvent
**1**	50	10	0.5	3.215	90.55	2.72	2.68	CH_2_Cl_2_, DMF
**2**	50	10	1.0	2.758	77.70	2.33	2.31	Acetone, DMF
**3**	50	10	2.0	3.100	87.32	2.62	2.60	CH_2_Cl_2_, DMF
**4**	50	10	3.0	3.273	92.19	2.77	2.75	CH_2_Cl_2_, DMF
**5**	50	10	4.0	3.287	92.58	2.78	2.75	CH_2_Cl_2_, DMF
**6**	50	12	0.5	2.600	72.95	2.19	2.00	DMF, DMAC
**7**	50	12	1.0	2.776	78.20	2.35	2.32	Acetone, DMF
**8**	50	12	2.0	3.060	86.20	2.59	2.55	Acetone, DMF
**9**	50	12	3.0	3.300	90.97	2.73	2.71	CH_2_Cl_2_, DMF
**10**	50	12	4.0	3.282	92.46	2.77	2.76	CH_2_Cl_2_, DMF
**11**	50	16	0.5	2.672	75.28	2.26	2.22	DMF, DMAC
**12**	50	16	1.0	2.583	72.77	2.18	2.10	DMF, DMAC
**13**	50	16	2.0	2.821	79.46	2.38	2.36	Acetone, DMF
**14**	50	16	3.0	3.013	84.88	2.55	2.52	Acetone, DMF
**15**	50	16	4.0	3.131	88.18	2.65	2.64	CH_2_Cl_2_, DMF
**16**	100	10	0.5	2.826	79.61	2.39	2.35	Acetone, DMF
**17**	100	10	1.0	3.255	91.68	2.75	2.78	CH_2_Cl_2_, DMF
**18**	100	10	2.0	3.325	93.66	2.81	2.80	CH_2_Cl_2_, DMF
**19**	100	10	3.0	3.297	92.88	2.77	2.76	CH_2_Cl_2_, DMF
**20**	100	10	4.0	3.227	90.90	2.73	2.70	CH_2_Cl_2_, DMF
**21**	100	12	0.5	2.5691	72.37	2.17	2.00	DMF, DMAC
**22**	100	12	1.0	3.239	91.24	2.74	2.71	CH_2_Cl_2_, DMF
**23**	100	12	2.0	3.237	91.18	2.74	2.71	CH_2_Cl_2_, DMF
**24**	100	12	3.0	3.289	92.65	2.78	2.75	CH_2_Cl_2_, DMF
**25**	100	12	4.0	3.297	92.87	2.79	2.76	CH_2_Cl_2_, DMF
**26**	100	16	0.5	2.508	70.65	2.12	2.10	DMF, DMAC
**27**	100	16	1.0	3.213	90.51	2.72	2.69	CH_2_Cl_2_, DMF
**28**	100	16	2.0	3.100	86.47	2.59	2.56	Acetone, DMF
**29**	100	16	3.0	3.233	91.08	2.73	2.70	CH_2_Cl_2_, DMF
**30**	100	16	4.0	3.212	90.49	2.72	2.70	CH_2_Cl_2_, DMF
**31**	200	10	0.5	3.246	91.44	2.74	2.71	CH_2_Cl_2_, DMF
**32**	200	10	1.0	3.331	93.82	2.82	2.82	CH_2_Cl_2_, DMF
**33**	200	10	2.0	3.205	90.27	2.71	2.70	CH_2_Cl_2_, DMF
**34**	200	10	3.0	3.263	91.90	2.76	2.72	CH_2_Cl_2_, DMF
**35**	200	10	4.0	3.142	88.51	2.66	2.65	CH_2_Cl_2_, DMF
**36**	200	12	0.5	3.111	87.62	2.63	2.60	Acetone, DMF
**37**	200	12	1.0	3.285	92.55	2.78	2.75	CH_2_Cl_2_, DMF
**38**	200	12	2.0	3.179	89.55	2.69	2.66	CH_2_Cl_2_, DMF
**39**	200	12	3.0	3.294	92.78	2.78	2.77	CH_2_Cl_2_, DMF
**40**	200	12	4.0	3.287	92.60	2.78	2.77	CH_2_Cl_2_, DMF
**41**	200	16	0.5	2.710	76.26	2.29	2.26	DMF, DMAC
**42**	200	16	1.0	3.295	92.80	2.78	2.79	CH_2_Cl_2_, DMF
**43**	200	16	2.0	3.122	87.94	2.64	2.63	CH_2_Cl_2_, DMF
**44**	200	16	3.0	3.231	91.01	2.73	2.72	CH_2_Cl_2_, DMF
**45**	200	16	4.0	3.246	91.44	2.74	2.71	CH_2_Cl_2_, DMF
**46**	300	10	0.5	3.243	91.35	2.74	2.73	CH_2_Cl_2_, DMF
**47**	300	10	1.0	3.057	86.12	2.58	2.53	Acetone, DMF
**48**	300	10	2.0	3.078	86.70	2.60	2.59	Acetone, DMF
**49**	300	10	3.0	3.167	89.21	2.68	2.67	CH_2_Cl_2_, DMF
**50**	300	10	4.0	3.126	88.05	2.64	2.61	Acetone, DMF
**51**	300	12	0.5	3.284	93.84	2.82	2.80	CH_2_Cl_2_, DMF
**52**	300	12	1.0	3.191	89.90	2.70	2.70	CH_2_Cl_2_, DMF
**53**	300	12	2.0	3.177	89.49	2.68	2.66	CH_2_Cl_2_, DMF
**54**	300	12	3.0	3.261	91.84	2.76	2.75	CH_2_Cl_2_, DMF
**55**	300	12	4.0	3.213	90.52	2.72	2.70	CH_2_Cl_2_, DMF
**56**	300	16	0.5	3.171	89.29	2.68	2.65	CH_2_Cl_2_, DMF
**57**	300	16	1.0	3.221	90.73	2.72	2.71	CH_2_Cl_2_, DMF
**58**	300	16	2.0	3.240	91.27	2.74	2.72	CH_2_Cl_2_, DMF
**59**	300	16	3.0	3.170	89.30	2.68	2.67	CH_2_Cl_2_, DMF
**60**	300	16	4.0	3.050	85.91	2.58	2.57	Acetone, DMF

To establish the generality of this method, a series of experiments have been performed under the same previous conditions except the catalytic amount of ferric perchlorate hydrate was (100 mg) (Samples **16**–**30**) (Table 1). After 0.5 h reaction time in different quantities of acetic anhydride, a sluggish acetylation reaction was occurred with low yield (79.61, 72.37 and 70.65%) (Samples **16**, **21** and **26**, respectively), whereas the other products were acetylated in high product yields (90.49 to 93.66%). In the case of 10 mL of acetic anhydride and from 1 to 2 h, the product yield began to increase. After just two hours at room temperature in 10 mL of acetic anhydride, we were able to obtain the highest product yield of cellulose tri-acetate (Sample **18** in 93.66% yield) (DS_exp_, 2.81), while with increasing time to 3 or 4 h, the hydrolyses occurred and the product yield decrease from 93.66 to 90.90% (Samples **18**–**20**). In the case of 12 mL of the acetic anhydride, the product yield began to increase from 91.24 to 92.87% by increasing the time from 1 to 4 h, so the reaction time has found to be an effective effect in Samples **22**–**25**) (Table 1). With increasing the acetic anhydride to 16 mL, the acetylation reaction takes one hour to give 90.51% yield (Sample **27**), then after that the product yield was not much affected by the passage of time from 1 to 4 h. From the above mentioned results, we have conclude that the reaction yield did not affected by changing the amounts of acetic anhydride from 10 to 16 mL while it strongly affected by the catalytic amount of ferric perchlorate hydrate (100 mg) (Table 1).

We investigated another catalytic amount of ferric perchlorate mediated by acetic anhydride after the success of this reaction. Indeed, 200 mg ferric perchlorate in various amounts of acetic anhydride (10, 12 and 16 mL) with different time reactions (0.5–4 h) successfully converted MCC to acetylated cellulose in 76.26 to 93.82 percent yield, demonstrating ferric perchlorate's catalytic capacity and impact (Samples **31**–**45**) (Table S1). A quick acetylation occurred with an excellent yield (93.82%, DS_exp_ 2.82) after a relatively short reaction time of 1 h in 10 mL of acetic anhydride (Sample **32**). Despite the longer reaction time, the product yield fell from 90.27 to 88.51% (Samples **33**–**35**). Using a higher volume of acetic anhydride (12 and 16 mL) in different reaction periods (0.5 to 4 h) yielded no important results (Samples **36**–**45**) (Table 1). To determine the standard experimental protocol, we used 2 g of MCC and treated it with AC_2_O (10, 12 and 16 mL) at room temperature in the presence of ferric perchlorate (300 mg) for various reaction times (0.5–4 h) (Table 1) (Samples **46**–**60**). The best acetylation yield was carried out using of 300 mg of Fe(ClO_4_)_3_·xH_2_O in 12 mL of AC_2_O for 0.5 h at room temperature to give Sample **51** in 93.84% yield (DS_exp_, 2.82). The fastest formation of cellulose acetates with excellent yield at room temperature was the most important finding of the current methodology. The functions of reaction times and the amount of the reagent AC_2_O are limited, so they will not have much of an impact on product yield, whereas the obvious effect was due to the different quantities of ferric perchlorate used in the various reactions. This method of making cellulose acetate is thought to be one of the simplest in terms of reactant amounts, and the reaction takes place at room temperature. The solubility test for the prepared samples showed that all samples with DS higher than 2.6 are soluble in CH_2_Cl_2_ and DMF while the samples with DS between 2.3 and 2.6 have a good solubility in acetone and DMF, which is very important from the industrial point of view (Table 1). This results of solubility in acetone is very important for the industrial production of acetone-soluble cellulose acetate for different commercial applications.

### Fourier transform infrared spectroscopy (FTIR)

**Figure 1:**
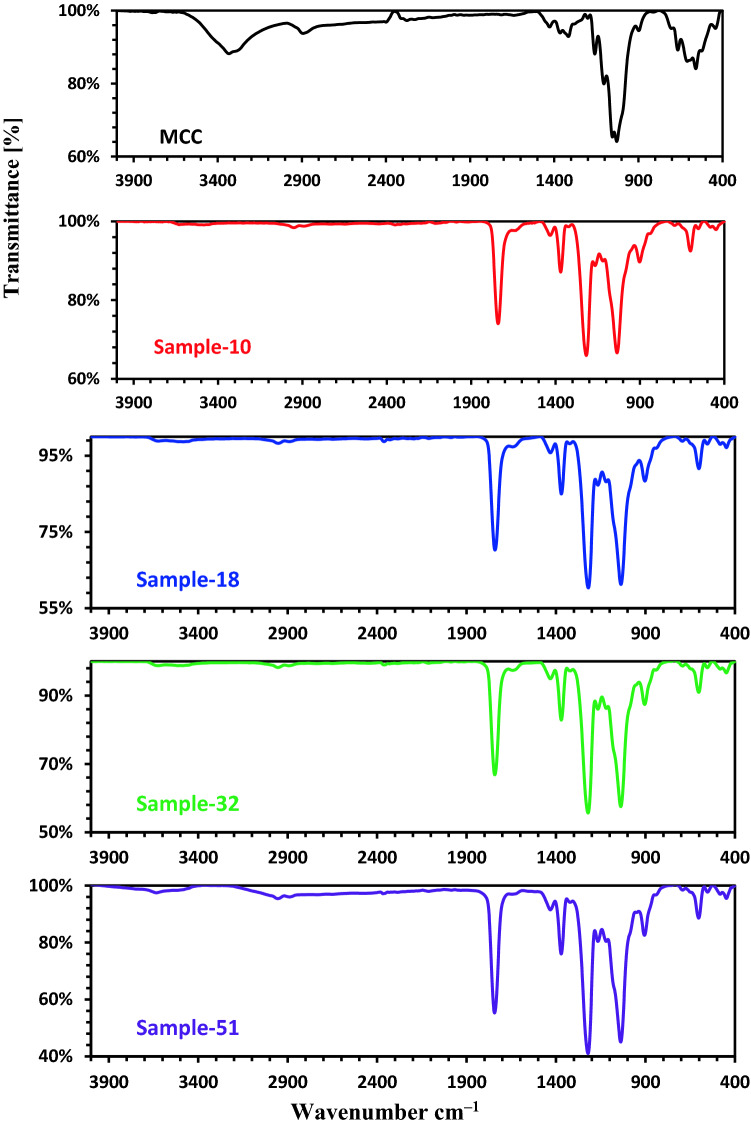
FTIR analysis of samples **10**, **18**, **32**, and **51**.

FTIR was used to analyze the microcrystalline cellulose and the prepared cellulose acetate. Figure 1 displays the FTIR analyses of cellulose acetates (Samples **10**, **18**, **32**, and **51**) and microcrystalline cellulose. The absorption band at 3388.93 cm^–1^ in the FTIR of microcrystaline cellulose is due to OH vibration^7,8,12,20,22^. The FTIR spectra in Fig. 1 display several distinct functional groups after the acetylation process. The key feature absorption bands appeared at (1741–1748 cm^–1^) which were assigned to carbonyl (C=O) as well as a diminishing in the strength of the band at 3388.93 cm^−1^ which was attributed to OH vibration, and the peak at (1365–1372 cm^–1^) was attributed to (C–H) in acetyl group. Furthermore, the sharp absorption peak at (1212–1222 cm^–1^) has been attributed to carbon–oxygen (C–O) stretching in a –O–(C=O)–CH_3_ group. C–O stretching in C–O–C linkages of cellulose, hemicellulose, and lignin was allocated a broad band at (1031–1038 cm^–1^)^21,23^.

### ^1^H-NMR spectrum

**Figure 2:**
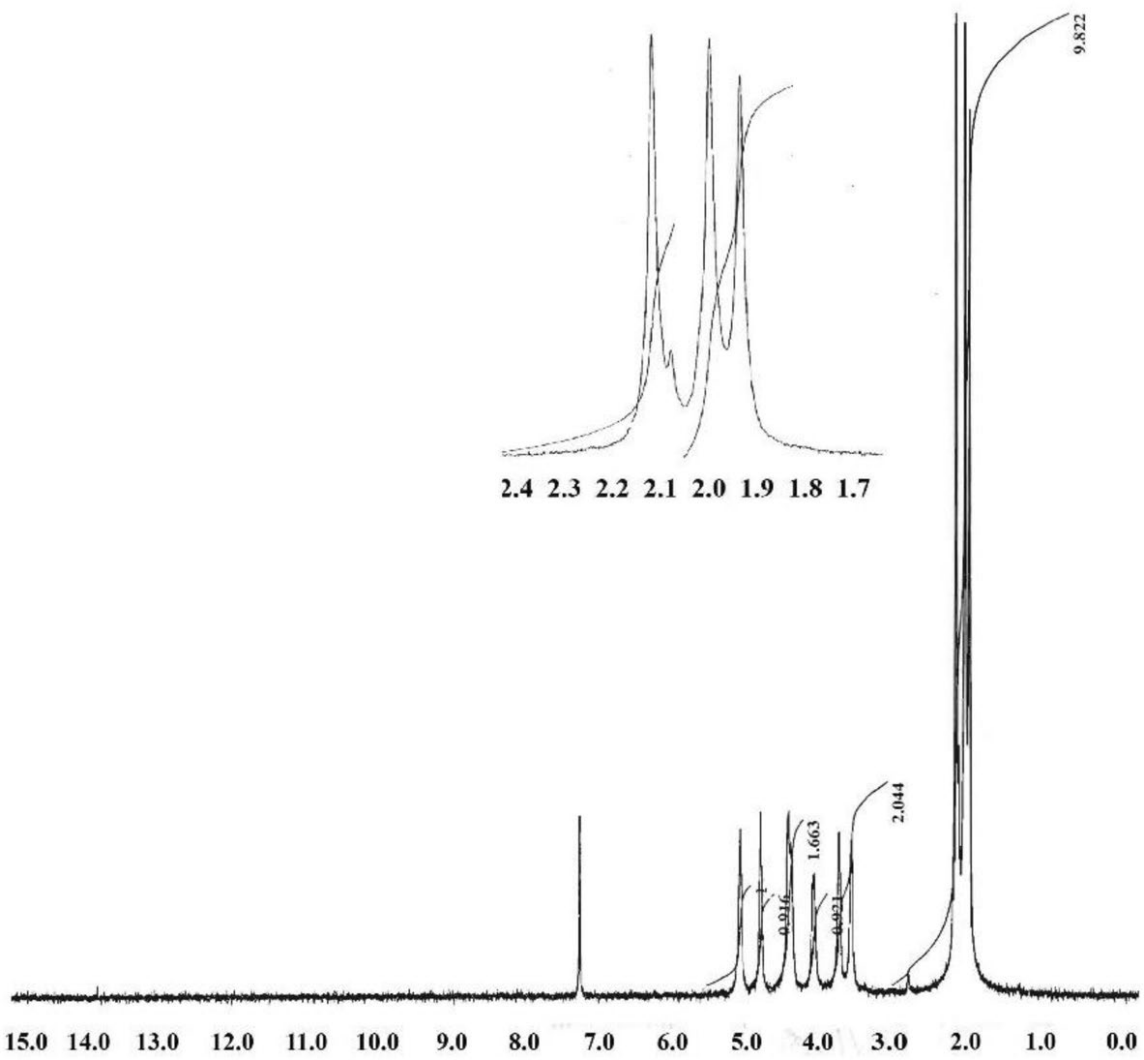
Cellulose tri-acetate 1H-NMR spectrum (DS = 2.82), Me stands for methyl protons in the acetyl group, H for anhydroglucose protons, and subscripts for Me or H positions on the anhydroglucose.

Figure 2 represents the ^1^H-NMR analysis of acetylated cellulose (Sample **32**) in 10 mL AC_2_O with 200 mg ferric perchlorate hydrate (Fe(ClO_4_)_3_·xH_2_O) as a catalyst and stirring at room temperature for 1 h. Two signal clusters can be seen on the hydrogen atoms (Fig. 2). The resonance of seven anhydroglucose protons were found at (= 3.53–5.06 ppm), while the three methyl protons of the acetate group were found at δ 1.93–2.12 ppm) (Fig. 2)^7,8,12,20–23^. The reaction product (Sample **32**) had a DS value of 2.91, which was derived from the ^1^H-NMR spectrum which confirmed the results obtained from the DS_Exp_ and DS_FTIR_. ^1^H-NMR spectrum proved that the formation of cellulose tri-acetate in Sample **32** is obvious.

### Gel permeation chromatography (GPC)

GPC was used for evaluating the degree of polymerization, weight average molecular weight (Mw), molecular weight distribution (MWD), Number average molecular weight (Mn), z average molecular weight (Mz), and the polydispersity index (Mw to Mn (PDI)) of prepared cellulose acetate. The polydispersity index and molecular weight averages are thus very useful in revealing the width of a polymer distribution. The molecular weight of a polymer-based product is an important property to know. A bell curve represents a molecular weight distribution, with the high end indicating high molecular weight and the low end indicating low molecular weight. The presence of a broad molecular weight distribution peak indicates that the sample is highly polydisperse, containing a variety of molecules of varying molecular weights. A sharp molecular weight distribution peak indicates that a monodisperse sample has mostly one small molecular range. Table 2 shows that as the catalyst concentration rises from 50 to 300 mg, the weight average molecular weight (Mw) and degree of polymerization (DP) also decrease. The DP and Mw did not changed substantially when the reaction time was increased from 0. 5 to 4 h or when the volume of acetic anhydride used was increased from 10 to 16 mL. Also, in this research, it was observed that, all the studied samples using GPC had a small polydisperses (Table 2).

**Table 2:** GPC analysis of selected samples of cellulose acetate prepared using different amount of ferric perchlorate catalyst and different amount of acetic anhydride.

Sample no.	Mn (g/mol)	M_W_ (g/mol)	M_Z_ (g/mol)	PDI	DP
**5**	12,061	45,280	116,825	3.754	167
**10**	11,788	46,362	116,362	3.933	174
**15**	13,257	51,227	135,354	3.864	201
**18**	13,974	50,895	134,143	3.642	189
**25**	7902	31,406	93,652	3.974	117
**29**	10,173	37,700	113,978	3.706	144
**32**	8041	25,936	58,973	3.225	96
**39**	11,358	33,793	76,444	2.975	127
**42**	6831	24,139	64,857	3.534	90
**46**	4249	11,901	33,891	2.801	45
**51**	5416	14,936	36,104	2.780	55
**56**	8798	25,467	62,149	2.895	98

The DP calculated values were calculated and normalized depending on the DS as previously reported^7,8^.

*Mn* number average molecular weight, *Mw* weight average molecular weight, *Mz* z average molecular weight, *PDI* polydispersity index, *DP* degree of polymerization.

### Thermal stability analysis

**Figure 3:**
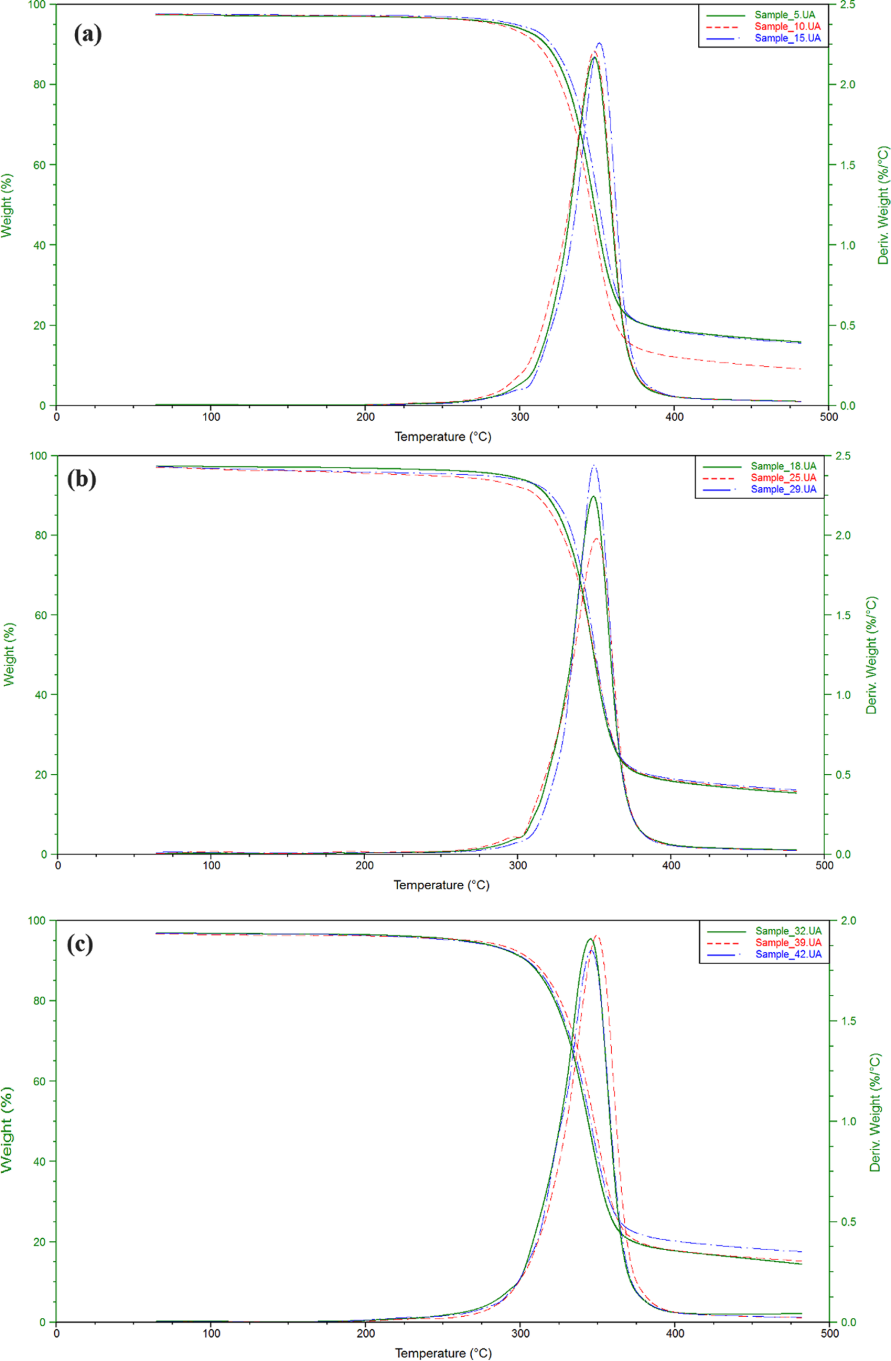

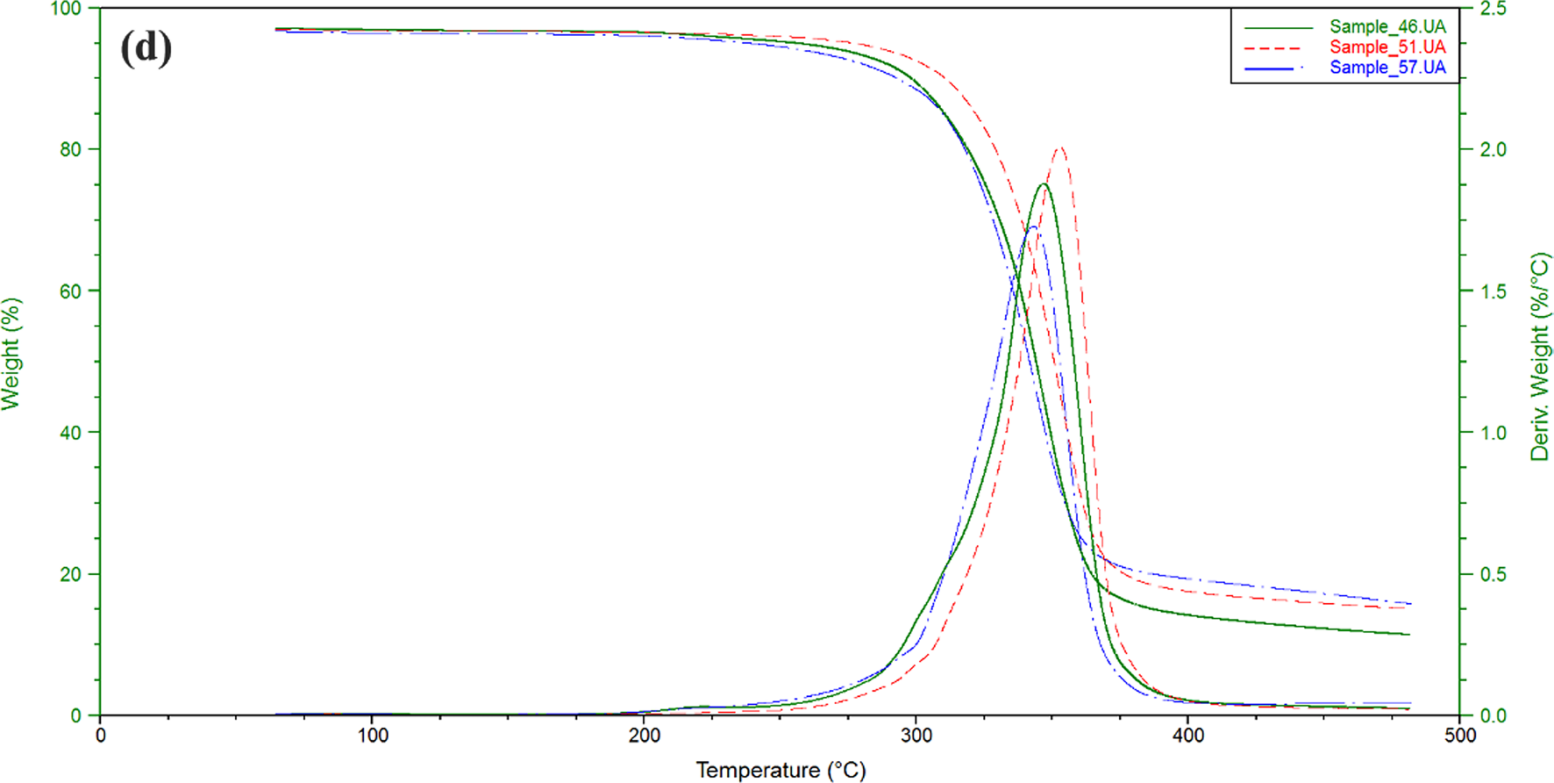
TGA and DTA analysis of (**a**) Samples **5**, **10**, **15**; (**b**) Samples **18**, **25**, **29**; (**c**) Samples **32**, **29**, **42**; (**d**) Samples **48**, **51**, **57**.

**Figure 4:**
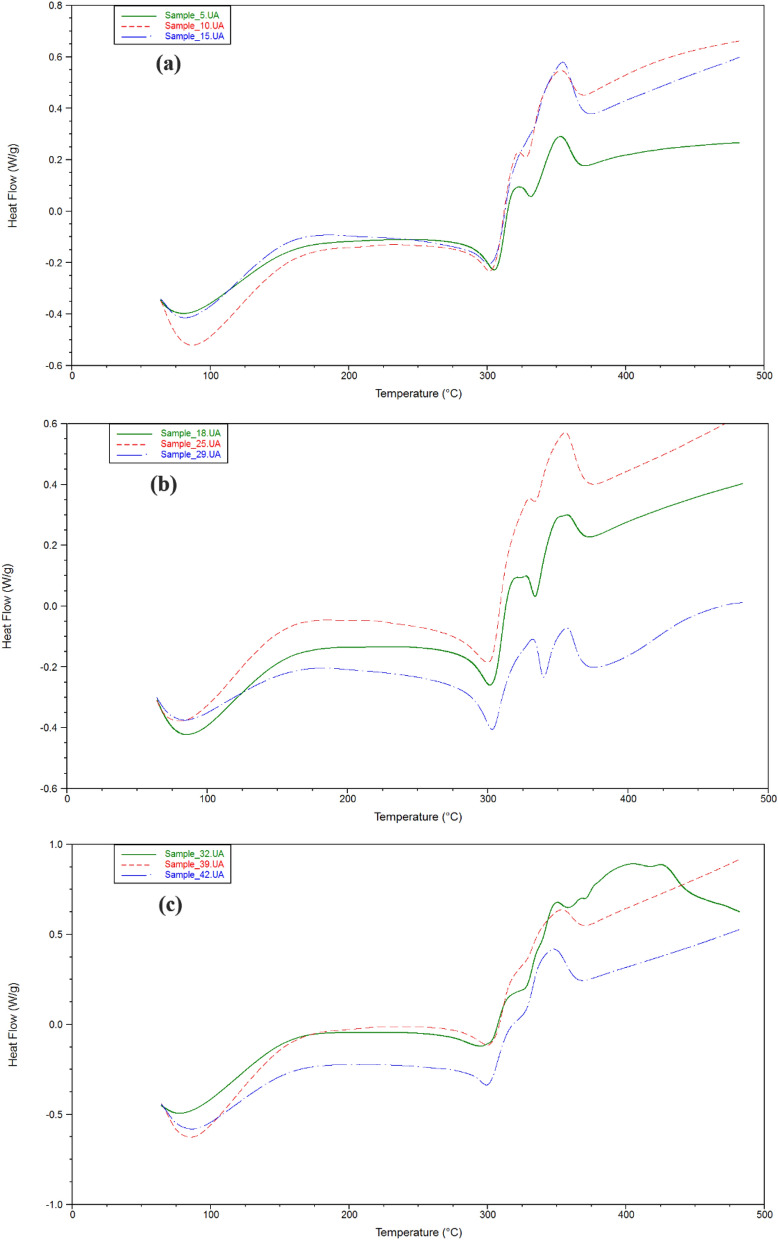

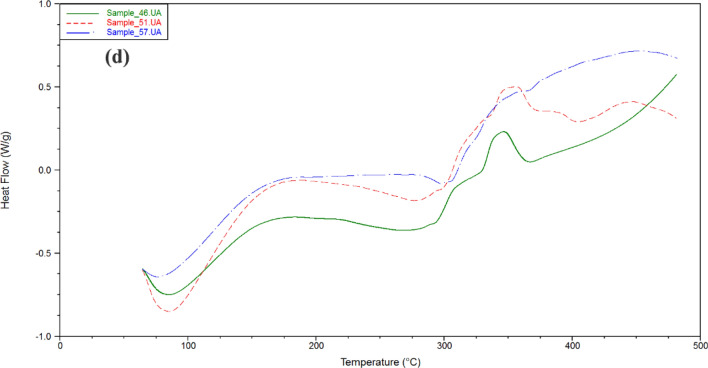
DSC analysis of (**a**) Samples **5**, **10**, **15**; (**b**) Samples **18**, **25**, **29**; (**c**) Samples **32**, **29**, **42**; (**d**) Samples **48**, **51**, **57**.

The thermal degradation of cellulose acetate has been studied by thermogravimetric analysis (TGA). Also the activation energy of decompostion of cellulose acetate has been determined from differential thermal analysis (DTA). Figure 3 showed dynamic TGA and DTA curves of cellulose acetate products with heating rate of 5 °C/min under 100 mL/min flow of N_2_. The thermal degradation of the prepared cellulose acetate has three series of degradation distinct zone. Initially, dehydration process was occurred at 100 °C due to the evaporation of bonding water on cellulose acetate in which depending on the hypophlicity of the cellulose acetate. However, there has not significant weight-loss at 100 °C. Then, the second zone has a rapid loss in weight due to the decompsition of cellulose acetate equal to (79–88%). Obviously, the temperature of decompostion of all examined cellulose acetates was founed to be approxmatily from 310 to 375 °C. The decomposition rate gradually decreases to a constant weight reflecting carbonization in the final region. The active temperature corresponding to maximum degradation rates (Tp) for the highest molecular weight cellulose acetate (15) is found to be 351.33 °C, while that for the other cellulose acetate products was (343.05–352.76 °C). Figure 4 shows DSC curves of cellulose acetate samples. An exodothermic peak was observed at around 100 °C for prepared cellulose acetates due to crystalization of water, this exodothermic event is in agreement with that observed in the TG analysis^29^. Another two exothermic peaks are observed around 310 and 330 °C assigned to prepared cellulose acetates crystallization. The broad enothermic peak around 360 °C can be assigned to melting of the crystalline regions of cellulose acetates^7,8,12,20–23^.

### Comparison the present work with some of the previous work

Different catalysts and methods have been reported for the synthesis of cellulose acetate with different degree of substitution. We previously reported different new catalysts for the synthesis of cellulose acetate using different reaction methods. The method reported in this work is superior in comparison with the literature work since the proposed reaction method is occurred at room temperature and in relatively short time. Also the high yield obtained in the present work is higher than many reported methods and comparable to the others (Table 3).

**Table 3:** Comparison between the yields of cellulose acetate using different catalysts.

No	Catalyst	Reaction Temp	Yield %	References
1	Fe(ClO_4_)_3_	25 °C	94.00	This work
2	NIS	140 °C	71.83	^20^
3	FeCl_3_	MW irradiation	90.58	^21^
4	I_2_	MW irradiation	100.00	^12^
5	ZnCl_2_	MW irradiation	95.83	^22^
6	MnCl_2_	140 °C	91.00	^23^
7	ZrOCl·8H_2_O	MW irradiation	~ 100.00	^24^
8	NaOH	100 °C	84.00	^30^
9	H_2_SO_4_	60 ± 5 °C	87.08	^31^
10	SO_3_H/PhSO_3_H-carbon	80 °C	48–77	^32^
11	H_2_SO_4_	25 °C	91	^33^
12	I_2_	80 °C	40–60	^34^

*MW irradiation* microwave irradiation in closed teflun cup as reported in leteratures.

## Conclusion

The objectives of this work was to find new efficient and inexpensive catalyst for successful esterification of microcrystalline cellulose under simple conditions with varying degrees of substitution (DS_EXP_ = 2.12–2.82). It has been demonstrated that using different methods of analysis such as FTIR spectrometer, ^1^H-NMR, GPC, and thermogravimetric analysis (TGA, DSC, DTA) proved that the di- and tri-cellulose acetate can be produced from microcrystalline cellulose using the proposed method. This esterification process often requires no solvent, a small amount of AC_2_O, and a Lewis acid catalyst (Fe(ClO_4_)_3_·xH_2_O), and it takes place at room temperature, in a short amount of time, and in a one-pot reaction system. With a DS_EXP_ value of 2.82, the higher yield of acetylated product is about 94%, and the reaction was completed in 1 h at room temperature. The acetylated product's DP (201) and MW (51,227 g/mol) values were found to be significantly higher in a small amount of catalyst (50 mg) than in a large amount of catalyst (100–300 mg). The presence of Lewis acid Ferric perchlorate hydrate catalyst is crucial for the direct conversion of microcrystalline cellulose to acetylated products, according to the findings. The effectiveness of ferric perchlorate hydrate as a catalyst has been verified.

## Data Availability

Data sharing is not applicable to this article.
